# Complexity, continuity and change: livelihood resilience in the Darfur region of Sudan

**DOI:** 10.1111/disa.12337

**Published:** 2019-04-04

**Authors:** Helen Young, Musa Adam Ismail

**Affiliations:** ^1^ Professor, Feinstein International Center, Friedman School of Nutrition Science and Policy Tufts University United States; ^2^ Associate Professor University of Zalingei Central Darfur State Sudan

**Keywords:** adaptation, conflict, Darfur, environmental variability, farming, livelihoods, maladaptive strategies, pastoralism, recovery, resilience, seasonality, Sudan, transformation

## Abstract

Darfur farming and pastoralist livelihoods are both adaptations to the environmental variability that characterises the region. This article describes this adaptation and the longer‐term transformation of these specialised livelihoods from the perspective of local communities. Over several decades farmers and herders have experienced a continuous stream of climate, conflict and other shocks, which, combined with wider processes of change, have transformed livelihoods and undermined livelihood institutions. Their well‐rehearsed specialist strategies are now combined with new strategies to cope. These responses help people get by in the short term but risk antagonising not only their specialist strategies but also those of others. A combination of factors has undermined the former integration between farming and pastoralism and their livelihood institutions. Efforts to build resilience in similar contexts must take a long‐term view of livelihood adaptation as a specialisation, and consider the implications of new strategies for the continuity and integration of livelihood specialisations.

## Introduction

The Darfur region of Sudan is a complex environment, characterised by extreme environmental variability and frequent droughts, a diverse ecology and a system of livelihood integration between farming and pastoralism that has existed for centuries. The region has a long experience of conflict, including wider civil wars, transnational conflicts, inter‐tribal conflicts and the Darfur conflict of the past 20 years, which prompted a huge international humanitarian aid programme from 2004. After more than a decade of protracted humanitarian action in Darfur, the government of Sudan and international actors shifted their attention to building resilience and early recovery of communities (UN, 2013).

Some resilience research focuses on theorising and the development of conceptual frameworks in order to draw policy and practice insights (Matyas and Pelling, 2015). However, there remains a dearth of empirical research that could help validate and advance theory in conflict and post‐conflict settings (Krampe, 2017). We seek to understand local perspectives on resilience, recovery and transformation in the context of extreme environmental variability and protracted conflict in order to inform decision‐making among a wide range of actors across scales, as well as policy and practice.

This article presents research findings from a study in the Darfur region of Sudan as part of the Building Resilience in Chad and Sudan (BRICS) programme. Our research interest is how farming and pastoralist livelihood systems manage production in the context of extreme environmental variability, and the long‐term impacts and transformations to their livelihood systems as a result of various shocks combined with political, economic and social processes of change. The study also considered the integration between livelihood strategies and systems, and prospects for peaceful co‐existence and sustainable futures.

The study is rooted in earlier scholarly work on traditional livelihood systems in Darfur and also theoretical considerations of socio‐ecological resilience, pastoralism and gender relations.

### Darfur livelihoods: a livelihood specialisation and cultural identity

Rain‐fed farming and pastoralism are the dominant agricultural production systems in the Darfur region (Haaland, 1991), and up to 30–40 years ago were highly integrated, as reflected by the exchange of a wide range of mutual benefits (Osman, 2012).

Farming and pastoralist systems are often associated with particular ethnic (tribal) groups. The term ‘nomad’ in the Darfur context is linked with both a cultural identity and the practice of camel and cattle pastoralism. Nomadic identity persists long after the people have settled or moved to the cities and no longer practise pastoralism. To avoid confusion, our interest is with the two main Darfur production systems: rain‐fed farming and pastoralism. By focusing on what people do, this article attempts to avoid stereotyping the people who practise farming and pastoralism as particular tribes, etc. Barth warned more than 40 years ago of the risks of viewing farming and pastoralism as distinct kinds of society:

*… we can focus not on two kinds of society, but—initially—on the total activities of a **region.** If we stop for a while thinking basically of **groups of people**, and think instead of **types of activity** we can then disaggregate the activities that take place in a region into some middle‐range sub‐systems which are **systems of production**, or ‘productive regimes'* (Barth, 1973; his emphasis).

This earlier view of production systems as part of a wider regional system is compatible with more recent thinking on the resilience of socio‐ecological systems (Adger, 2000; Folke, 2006; Bousquet et al., 2016). It is also compatible with the non‐equilibrium paradigm on dry lands development, which recognises that dry lands are characterised by extreme variability, especially precipitation, and are thus more accurately described as a non‐equilibrium environment rather than a single state of equilibrium (Behnke et al., 1993; Scoones, 1996). For production to be sustained in this context requires management strategies that deal with the extreme rainfall variability (Mortimore, 2009). This reflects the earlier work of Holling (1973), whose seminal reference on resilience focused attention on the management approach to sustaining productivity under conditions of extreme instability or variability (Walker and Cooper, 2011).

This systems view contrasts with much of the international humanitarian and media narrative on Darfur over the past 15 years, which has remained stuck in a more dichotomous view of Africans versus Arabs and farmers versus herders (Mamdani, 2009). Partly as a result of the conflict dynamics and the perceived political affiliation of particular tribes, this narrative continues to hold fast. Early on, this contributed to a narrowing of the view of the international community, which works predominantly with internally displaced people while largely ignoring the situation of some pastoralists and nomads, especially women.

While this article does not include a sub‐regional conflict analysis, it reflects the approach of Grawert (2008), which focuses on the way conflict has played out over time, the actors involved at different levels and shifts in power relations (in this study, over access to resources and the implications for the continuity of livelihood specialisations).

The integration between the two livelihood sub‐systems reflects their symbiotic relationship (Barth, 1973), whereby cultural, social and economic links span multiple levels, starting from individual producers and extending to the national and international level. Darfuri farmers and herders continue to be dependent on shared systems of natural resource use. This means the symbiosis and environmental cooperation between these two regional livelihood sub‐systems remains salient today (Abdul‐Jalil, 2008; Osman et al., 2013).

Another dimension of this research was the integration of women and gender into the study design, recognising the need to analyse gender inequalities and gendered livelihood relations if BRICS was to address the vulnerabilities of women (Bradshaw, 2015; Le Masson, 2015).

## Methodology

### Selection of communities, timeline and training

**Figure 1:**
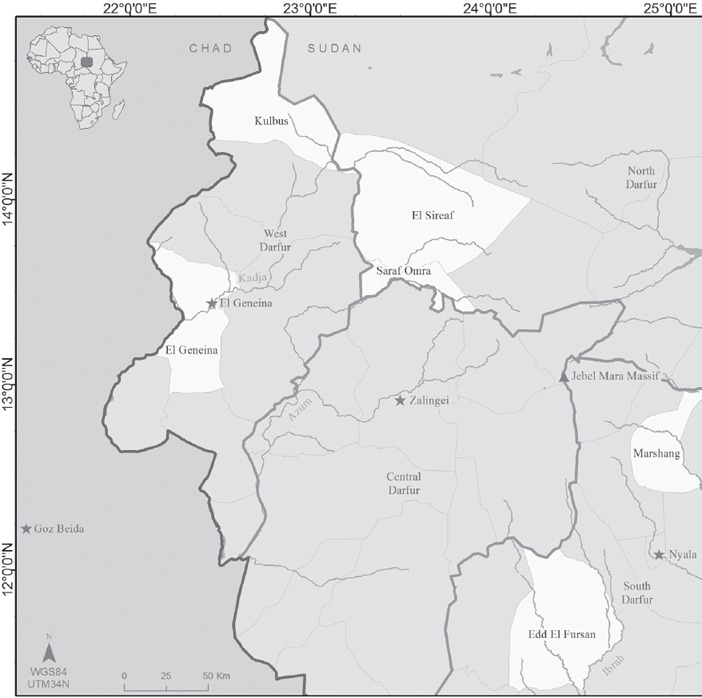
Map of the Darfur region highlighting where the study communities are located **Source**: authors.

A research team of 12 professionals drawn from local academic and government institutions collected and analysed data from two rounds of field visits and qualitative data collection in 11 communities in West, North and South Darfur in August 2016 and April 2017 (see Figure 1). The findings also draw on the results of a baseline survey completed in December 2016 (BRICS, 2016). Intensive training covered theory and concepts related to resilience and non‐equilibrium models of production, qualitative methods and practical field techniques.

### Qualitative tools

Qualitative research tools included a field workbook, with guidance and instructions for focus group discussions (FGDs) and key informant interviews (KIIs) and participatory techniques (timelines, seasonal calendars, resource mapping, proportional piling, matrices and Venn diagrams). The Tufts Internal Review Board (IRB) approved all research instruments and the Humanitarian Affairs Commission gave in‐country permissions. In total, the teams completed 80 FGDs with men and women separately, 75 KIIs, 21 household interviews and 18 meetings with leaders. This covered a total of approximately 1,005 individuals.

The outputs of participatory exercises and notebooks were recorded in Arabic and later professionally translated to English. Field notes and worksheets were imported into qualitative software (NVivo version 11) for review, coding and further analysis. An important stage of the analysis entailed subsequent team meetings and BRICS partner workshops in Khartoum and El Geneina to review findings and discuss their implications for short‐ and long‐term resilience to shocks and for current BRICS targeting and activities. The researchers produced four regional reports plus a synthesis of the lessons learnt and recommendations for in‐country programmatic review.

### Limitations

The wide geographic spread of communities across three states led to long travel times and complex logistical arrangements. Rainy season fieldwork was seriously impeded by flash flooding and blocked roads, and teams had to return later to complete the work.

## Study findings

### The study communities

All 11 communities were living in permanent settlements, with access to a nearby *wadi* (a seasonal watercourse). Five communities identified themselves as nomads, although they had permanent settlements some going back to the early 1990s, if not before. This process of nomadic sedentarisation accelerated following the Darfur conflict starting in 2003. Box 1 provides background on the 11 communities. Men and women FGDs developed community resource maps illustrating features and resources they considered important; the men's FGDs frequently captured the settlement and wider surroundings, whereas some women's FGDs focused on the settlement itself, with details of community institutions, including the mosque, sheikh's house and school. Across communities, these maps show a similar range of natural resources (water resources, forest, land for seasonal cultivation and rangeland).

All 11 settlements were located within easy reach of a *wadi* system (providing rainy season water and dry season shallow wells), allowing them access to different soil types, including more fertile alluvial soils close to the *wadi* and sandy (*goz*) soils for cultivation of rain‐fed crops. The alluvial soil of the dry river valleys or *wadis* was more highly valued, because ‘*it can yield high production and is also suitable for fruit plantations and for brick‐making*’ (FGD Doga), compared with *goz*.

All community maps included nearby forest resources, which in a few cases were off‐limits as women faced harassment from individual hostile herders (linked with the polarised relations between some farming and herding communities that were part of the wider Darfur conflict). More recently, the 2012 West Darfur National Forest Commission prohibition on cutting of trees has also restricted access to forest resources.

All but one village (El Beida in North Darfur) currently practise a combination of rain‐fed cultivation of *goz* soils and dry season cultivation of alluvial soils along the *wadi*.

The people of El Beida and El Kother (North Darfur) are former nomads who have settled in the past 15 years (El Beida) or recently relocated (El Kother) but continue to practise pastoralism with migration of herds. The maps of nomadic communities frequently included livestock corridors and also *fariq* (a temporary nomad camp), reflecting their pastoralist system.

Traditional institutions, such as the Native (tribal) Administration, are still present and continue to play a role in community life. In some communities, FGDs said that in the past the sheikh had had more authority to solve problems compared with the present. This was because the problems communities faced were more within his control and also because he was more able to coordinate with local government departments or seek interventions from other locality institutions, or to mobilise the community to protect natural resources for example (e.g. by opening fire lines to protect pasture and forests). In addition to tribal leaders, there now exist a range of other formal and informal community institutions, for example the Popular Committee, the Women's Association, the Youth Association and, of increasing importance in several areas, committees intended to promote farmer–herder relations and to address the problems of pre‐harvest crop damage and expansion of farms blocking corridors.

**Box 1. The 11 communities included in this study**

**North Darfur**

El Beida and El Kother communities are part of the nomadic El Waha locality and share a cultural identity as *aballa*—camel‐herding nomads. The people of El Beida first moved south to settle in El Beida in the 1980s following the famine and continue to herd camels in the manner of their ancestors. The El Kother community also migrated south following the 1980s famine, to Seraif locality but were displaced in 2011 as a result of the tribal conflict over gold‐mining and moved to El Kother.

**South Darfur**

The people of Um Sayala and Abu Rojo identify themselves as nomads, with many continuing to practise camel pastoralism. Both communities have more diversified livelihoods now compared with in the past. Hashaba, by contrast, is a more mixed community, including different tribes and groups that have relocated to the area since the 1960s. Hashaba livelihoods include rainy and dry season farming, with some people working on the farms of urban investors or in partnership agreements with the landowner.

**West Darfur: El Geneina locality**

In the past, Doga and Manzola livelihoods depended on cultivation of rain‐fed and dry season crops, with some livestock (cows, sheep and goats). They were severely affected and impoverished by the Darfur conflict, having lost their livestock early on and continuing to face difficulties accessing land for cultivation. Both communities were displaced in 2004. The Doga community resettled to its current area about 9 km from the original one, and continues to cultivate their original lands, but faces problems. The Manzola community has returned to its village but is able to access only a limited area to cultivate. The people of Telehaya were fully nomadic in the past and began to settle in the early 1990s; many continue to practise pastoralism but their herds are smaller and their mobility less extensive than before. Since settling close to a bustling market town, the Telehaya nomads have diversified their livelihood activities to include farming and trade.

**West Darfur: Kulbus locality**

Livelihoods in Goshosh and Bir Taweel were traditionally agro‐pastoralism: a mix of mainly pastoralist livestock‐keeping and some rain‐fed farming. In the past decade, the balance of activities has shifted from livestock to farming, because of livestock looting and the increasing importance of dry season agriculture. They depend mainly on agriculture and say they have ‘*renounced nomadic life and become farmers*’ (Herder, Bir Taweel).

**Source**: authors.

### Managing variability: Livelihood specialisation and seasonality

Farming and pastoralism sub‐systems are seasonal by nature, with periods of intense work, with all other livelihood activities fitting around the livelihood specialisation. Across the 11 communities, respondents described the same five distinct seasons and their characteristics in detail (Box 2). Some communities emphasised the seasonal changes linked to rain‐fed cultivation (Manzola and Doga, West Darfur); others described their changing environment in relation to the needs of livestock for food and water, and where these could be found, again revealing their specialised knowledge.

The extreme inter‐annual rainfall variability gives rise to fluctuating harvests, with good and bad years. Even within a localised area, production can be very uneven, with some farms receiving adequate rain while others might fail.

Thus, communities are very familiar with extreme rainfall variability, including the possibility of a late start to the rains; gaps of several days without rain; heavy downpours or floods; and the uneven or patchy distribution of rains. Both farming and pastoralist communities could describe in detail how rainfall variability, high winds and excessive heat might affect the quality and productivity of their crops or the health of their livestock. They also described their own specialist practices for managing environmental variability.

**Box 2. The five Darfur seasons**

The climatic year starts in anticipation of the first rains, or *rushash*, which are important for farmers and herders for different reasons. The timing of *rushash* varies from year to year, and also because the rains start first in the south and then gradually advance north. Thus, El Geneina communities reported *rushash* as from April to May while further north in Kulbus *rushash* is from May to June.

The established rainy season, or *kharif*, follows *rushash* and lasts about three months. With the rains, temperatures drop, humidity increases and there are cool breezes. There is rapid growth of vegetation, pasture is available, including a greater diversity of grasses, and trees turn green. Drinking water from *wadis* and surface water is widely available. Insects and pests appear and increase, and certain diseases appear or become more prevalent, including malaria, diarrhoea and bronchitis.

*Kharif* is followed by *deret*, with the end of the rains, increasing temperatures and reducing humidity, allowing crops to mature prior to harvesting. *Deret* is a time of plenty and was described as the ‘*master of seasons for farmers and herders for its abundance of good things*’ (FGD 1, Hashaba). The following dry season is split into two periods, starting with *shita*, the cool dry season, and *seif*, the hot dry season.

*Shita* is important for the ripening of dry season crops and early vegetables such as green mellow, watermelon and tomato. *Seif* is the time of hot weather, rising temperatures and an ‘inactive’ or ‘stagnant’ wind. There is a widespread lack of water for people and animals, and they must depend on a limited number of permanent sources, the quantity and quality of which continue to decline with increasing use. With the high temperatures during *seif* some diseases emerge, including meningitis.

Variability in the start and end dates of the rainy season are widely acknowledged, as well as extreme variability in the spatial and temporal distribution, and intensity of the rains, throughout the season. Many respondents felt that weather extremes, including drought and floods, were increasing in the region, although this was not apparent in the historical timelines.

**Source**: authors.

**Figure 2:**
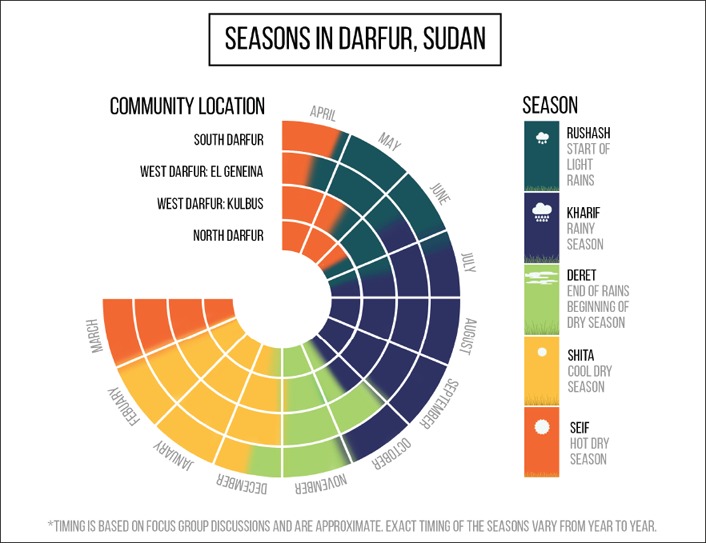
Five Darfur seasons by locality and approximate month **Source**: authors.

Part of pastoralist specialisation is the seasonal migration of cattle and camel herds. During *rushash*—the start of the rains—the herds are in the south, and some herds may move further south at this time to meet the rains earlier than if they remain where they are. Herds then move northwards with the advancing rains and greening‐up of pasture, until they reach the more northern grazing areas, where they stay moving around until the end of the rainy season. As water and sometimes pasture become more limited in the north, the herds return south where there is more plentiful pasture and fodder and permanent water sources to keep them going through the dry season until the next rains. The pull factor for migration north is the better pasture and breeding conditions for livestock; at the same time, this enables herds to avoid the challenges of mud and flies present in the far south at this time. Another factor farmers mentioned was that this migration north keeps the animals away from the farms, thus reducing risk of crop damage during the agricultural season.

In the past, camel and cattle herds travelled distances of 400–600 km between northern rainy season grazing areas and dry season grazing areas further south. This seasonal movement loosely tracks known routes or corridors (*murhal*), although the width of these varies and there may also be tributaries. At times, the livestock routes become narrower, enabling herds to pass through farming zones without problems; at other times, they may be blocked by farms, or alternatively broaden out to cover vast areas. After the rainy season, the return migration southwards coincides with the post‐harvest period, potentially allowing pastoralist herds to graze post‐harvest crop residues and fertilise farmers’ fields, and providing opportunities for exchange between farmers and herders. Both farming and nomadic communities had memories of these mutual benefits and the exchanges that used to happen, although almost universally this system is no longer practised in the communities included in this study (see Discussion). Farming communities also keep livestock; however, numbers have greatly reduced as a result of looting and insecurity linked to the wider conflicts. In our farming communities (Goshosh, Bir Taweel, Manzola and Doga), only a small number of goats and sheep were kept, and these were herded in the local vicinity. In the past, livestock assumed a greater importance for farmers; for example, in Doga, according to a former dairy woman, breeding sheep and goats was an important activity for women linked to commercial sales of curdled milk and dairy fat.

Farming communities also recounted their practices for optimising production given the unpredictability of the rains. Two traditional practices to maintain production are shifting cultivation and distributing fields so as to reduce risk of rain failure. Shifting cultivation involves clearing uncultivated land and farming for a few years before moving on to clear a new piece of land, allowing the previous farmed area to regenerate. Respondents, with the exception of some older farmers in Kulbus locality, reported that this was no longer practised because of the shortage of cultivable land and changes in land tenure. Hence, farming is now continuous, with no fallow periods, and very few, if any, inputs. Crop rotation is commonly practised, diversifying crops over multiple seasons, as well as planting a range of different crops (maize, broad beans, millet and watermelon).

**Figure 3:**
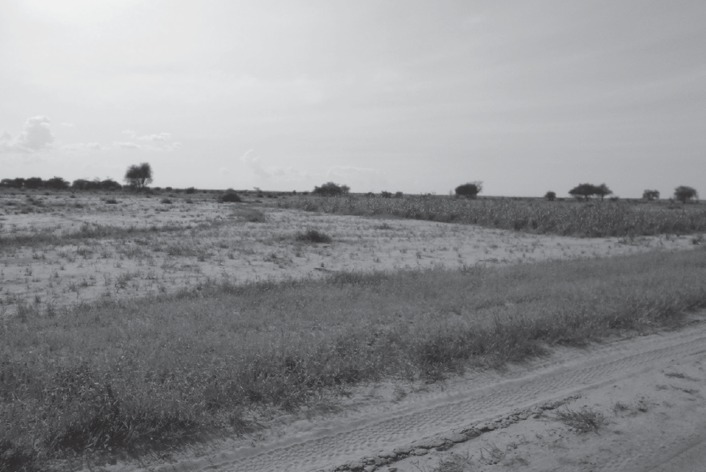
Example of crops from dry sowing of seeds (before rains start) compared with later plantings (more advanced crops are from dry sowing) near Goshosh **Source**: authors.

**Figure 4:**
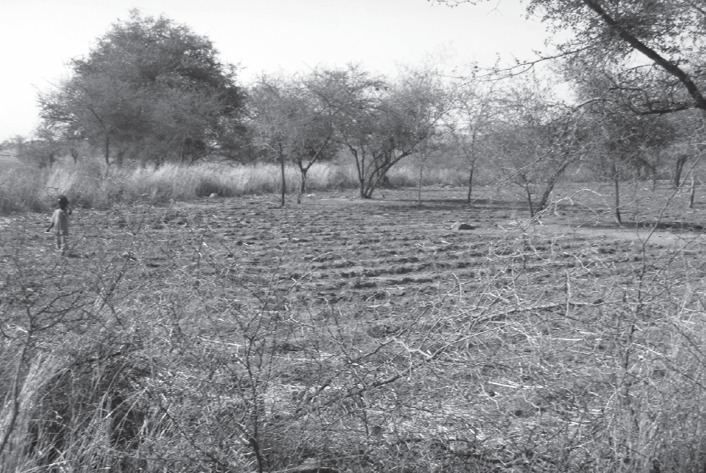
Use of contour ridges to conserve water for agriculture in farms near Manzola, May 2017 **Source**: authors.

Various practices are used to ‘catch the rains', or take full advantage of the rains when they do fall. These include early cultivation, as soon as the soil conditions are damp enough to allow germination (Manzola and Doga, El Geneina locality). Alternatively, in Kulbus locality, which has up to 100 mm less rainfall per annum, farmers traditionally practise dry sowing (*rinmail*): planting millet seeds in sandy soils before the rains start, which produces faster germination and better growth according to farmers, as shown in the example of crops sown at different times in Figure 3. Third, contour ridges or barricades are constructed to conserve water (Figure 4). If rains are delayed, farmers will replant their seeds, sometimes two or three times, in the hope of securing a harvest, albeit a smaller one because of the shorter season. Once seedlings are established, farmers will check which fields are doing better, and if necessary transplant weaker seedlings to fields in areas of better rainfall. Farmers in Manzola will also test the fertility of the soil with experimental planting. For example, with okra, they will first plant a small amount of seeds and test first whether they germinate in their plots; if successful, they will then plant a wider area.

Other strategies to sustain production include the diversification of crops over multiple plantings. Also, farmers who own livestock will keep these in corrals on the farms post‐harvest, moving them around until all their fields are manured. Farmers often stay for part of the season in shelters near their crops, to weed them and to protect them from trespassing livestock, etc.

### Managing change: Hazards, shocks and coping

Over the past 50 years, the study communities have been exposed to a continual stream of hazards and actual shocks, including persistent droughts (over consecutive years), floods, various conflicts and other shocks (pests, livestock epidemics, livestock looting and idiosyncratic shocks). Community elders developed historical timelines detailing the different shocks affecting their communities (see Table 1), and FGDs and household case examples recounted the losses and damage to their livelihoods from particular shocks, as well as their coping and adaptive strategies.

**Table 1:** Historical timelines of shocks for communities in Kulbus locality, West Darfur

Timeline	Shock
1972–1973	The *Sugu* famine
1981–1982	Chadian civil wars
1984	Famine–*Maga Ammi Nasheelaha* (Take Your Mother Famine)
1988	Severe flood
1990	Drought
1992	Pests—*al‐Shaw* (The Red Locust)
1995–1999	Tribal conflict
1997	Famine
2000	Conflict in Chad led to displacement in Bir Taweel
2002	Conflict and displacement
2003	Darfur insurgency
2005	Drought
2006	Cattle theft
2006	Scarcity of rain/drought
2007	Lack of rain and locust attack
2008–2010	Insecurity and road closure
2010	Border threats and increasing risk of livestock damaging crops
2010	Livestock theft
2012	Poor rainy season
2014	Agricultural pests
2015	Floods
2015	Scarcity of rain
2015–2016	Herder farmer conflict and blocked access to farms

**Source:** authors.

The nomadic communities of North and South Darfur reported a total of five or six droughts, while the farming communities of Kulbus and El Geneina in West Darfur reported a total of eight or nine incidents. This difference may in part be a result of regional differences in rainfall patterns; more likely, it reflects their different strategies for managing droughts.

Droughts of one season's duration are patchy in their impact, only affect certain communities and were generally not considered a major problem unless combined with other problems or shocks. For example, the 1991 drought in North and South Darfur was exacerbated by the change in the national currency (from Sudanese pounds to Sudanese dinar), which particularly affected nomads, as they missed the deadline for exchanging their old currency. Furthermore, when droughts persist over consecutive calendar years, or combine with other shocks, such as the rinderpest epidemic of cattle during the great famine of 1982–1984, the combined impact is multiplied and recovery takes far longer.

The two most severe famines in living memory were associated with persistent drought (1972–1973 and 1982–1984). They were identified by all communities and their devastating effects were well remembered:

*In their lifetimes they had never seen such a crisis. People died, and men fled and left their children and wives. Animals died, and people watched others die in their hands and there was nothing they could do. All the livestock came to an end. Families were separated and have not reunited to this day* (elders from Goz Deiga, Kulbus Locality describing the 1980s famine).

From the accounts of community elders, the 1982–1984 droughts marked the beginning of pivotal changes in livelihood systems. For farming communities, this led to migration (displacement in search of aid, increasing labour migration and, for many more northern agro‐pastoralist communities, southwards migration and relocation).

For nomads, the experience of two epic famines (1972–1973 and 1982–1984) in close succession was epoch‐making. In South Darfur, the 1982–1984 famine was exacerbated by a rinderpest epidemic, reported in all three south Darfur communities. Together, the series of droughts and livestock epizootics resulted in catastrophic losses of cattle in the south. Elders from nomadic communities described how former wealthy nomadic families became impoverished as a result of loss of livestock, while also acknowledging that long‐distance livestock migration saved many livestock. This seminal experience prompted many pastoralist households to diversify their livelihoods, in part because they saw the more rapid recovery of farmers following the 1986 and 1988 good harvests. This prompted some nomadic households to partially settle, with women nomads beginning to cultivate in the rainy season and copying the activities of their farming neighbours, while men continued to move with and manage their pastoralist herds. This pattern of diversification into farming was evident among the nomadic communities in North and South Darfur, as well as in Telehaya and Bir Taweel in West Darfur.

The impact of floods had been far less widespread than that of drought, especially persistent drought. Flash floods washed away topsoil and young crop, and flooding of dry riverbeds where livestock congregated caused many to drown. Elders noted that floods often occurred in years of very good harvests: while the valley area is flooded, higher areas of usually sandy soils are more productive than usual.

In addition, study communities have had to contend with multiple conflicts of different types, severity and duration, ranging from localised inter‐tribal conflicts; the wider Darfur conflict between Darfur rebel insurgents and the government counter‐insurgent forces that started in 2003/04; and Chad's cross‐border conflict. In the early 1980s, conflict within Chad between the Chadian government and opposition forces spilled over the border. In 2015, Chadian herdsmen caused problems for the people of Bir Taweel, preventing farmers from farming by blocking access to cultivable land and seizing some ploughs. More recently, there have been disputes between Tama farmers and Chadian (Bedeyat) herders over the farms on the western side of Goz Deiga.

All communities reported a range of inter‐tribal conflicts, often over access to natural resources, whether cultivable land, rangeland or control of traditional gold mining, and associated with loss of lives and livelihood assets, forced community relocation or displacement.^1^ The reorganisation of the tribal administration in West Darfur in 1995 triggered a major tribal (Arab Masalit) conflict, which had devastating effects. Tribal clashes in North Darfur starting in 2000 eventually led to a wave of resettlement of nomadic groups from Kutum to the El Kother area. Much later, in 2011, tribal conflicts over control of traditional gold mining in the Jebel Amer area led to the forced relocation of the El Kother community. All three South Darfur communities reported increasing inter‐Arab tribal conflict from 2006. Other South Darfur tribal conflicts, affecting Hashaba community, included the Gimir Beni Halba conflict, which continued up to 2013.

The most severe and widespread conflict affecting the West Darfur study communities was the wider Darfur conflict, pitting government counter‐insurgency forces against Sudanese rebels, which started in 2003, with widely reported attacks on civilians (de Waal, 2004; Flint and de Waal, 2005; Young et al., 2005). Armed clashes in the area of Goshosh and Bir Taweel were linked to armed robbery, looting of livestock, ransacking, burning and destruction of homes. In Goz Deiga, the people fled for their safety to Chad and returned home shortly after. Direct attacks also displaced others in Manzola and Doga, who displaced to camps near towns. The historic timelines of the nomadic communities did not refer to this period, as this conflict shock did not affect them directly.

As well as these specific conflict episodes, in West Darfur elders described periods of insecurity linked with general banditry and lawlessness; road closures and restrictions on mobility caused by nearby armed clashes; threats of livestock looting; and pastoralist livestock herds damaging standing crops. The latter was first reported in Goshosh, West Darfur, in 2010, but has since become a continuing risk, sometimes perceived by farmers as intentional on the part of herders.

In the household case studies, women and men often described idiosyncratic shocks, including for example death of a husband, migration or long‐term disappearance of a husband, divorce, chronic disease and death. These events had reduced the household labour potential and ratio of dependants to adult workers.

#### Coping with continuing risks

The endless stream of shocks affecting all communities over the past 50 years has contributed to an on‐going process of coping, including seeking ways to increase production and additional sources of food and income. Farming communities have expanded and intensified their agricultural production to include irrigated agriculture, inter‐cropping, dry season irrigated farms and gardens producing cash crops. Dry season agriculture and marketing of the cash crops grown has increased in particular, although this depends on household access to suitable land and water for irrigation. There has also been an intensification of land use in the immediate environs of former displaced communities, because of the security risks associated with farming beyond the village boundaries. Farmers explained that this had also contributed to shrinking farm size and reduced productivity.

The settled nomadic communities of North and South Darfur, and Telehaya in West Darfur, especially women, are attempting rain‐fed farming but, especially in South Darfur, they lack the skills and frequently rely on paid agricultural labour to work their farms. The pastoralists are also changing the composition of their livestock herds, from camel and cattle, which are traditionally favoured, to include sheep,^2^ which are valued for their quick economic returns in the context of a thriving regional and national market. A Telehaya herder explained that, among the settled nomads, sheep are preferred ’ *for their quick reproduction, decent prices, ease of herding and not being stolen*’ (herder, Telehaya). However, sheep are the species most vulnerable to disease. Consequently, sheep are the most costly species in terms of medical treatment and timely vaccination in case of emerging infectious diseases (Sulieman and Young, 2018).

Labour migration is a long‐standing dry season strategy for some communities (Grawert, 1992; Pyle, 1993). Forced migration, driven by conflict, has been a widely reported feature of the Darfur conflict. As one former displaced women explained, *'After our displacement we came to these livelihoods not as a choice but to survive,'* thus illustrating the restrictions on individual households’ capacity and the loss of human agency associated with insecurity and changing relations (see below).

The nomadic communities are better off than the farming communities in terms of their livelihood asset ownership (livestock, access to fields and other wealth indicators) based on the 2015 baseline data (BRICS, 2016) and the qualitative data. However, from the wealth ranking exercise, the poorer wealth groups in the farming communities and in the nomadic communities are remarkably similar; both practise the same marginal low‐return activities and have very few livelihood assets. This indicates there is far greater inequality within the nomadic communities compared with the farming and former displaced communities. This finding reveals the hidden vulnerability within apparently better‐off nomadic communities, especially among nomadic women‐headed households that have no livestock or access to land and must survive on marginal day labour activities.

Trade and markets are crucially important to all of the above livelihood activities, whether trade in livestock, crops or natural resources, or employment or casual work in relation to these commodities. As a result, the proximity of a community to a regional market clearly matters, and makes a huge difference to livelihood opportunities and subsequent prosperity. For example, Telehaya, close to the state capital El Geneina, and Hashaba, close to Nyala, capital of South Darfur, has far more market‐oriented economic opportunities than other, more distant, communities.

There are several newer forms of diversification, many linked with the emerging war economy and conflict, such as young men joining the armed forces or militia or increased trade connected to servicing the needs of the internally displaced persons camps. Brick‐making is present almost everywhere, with poorer people supplying the labour. Artisanal gold mining in North Darfur acts as a magnet to men from communities across West Darfur as well as North Darfur.

#### The gender‐specific division of labour

Although both women and men can undertake most farm‐related tasks, it is women who carry the largest farm work burden in both farming and nomadic communities. Among the settled nomads in Telehaya, it is women who work in the farms during the rainy season, with men more likely to be involved in dry season cultivation of cash crops. Women and men both highlighted the importance of trade because of the proximity to El Geneina town. One Telehaya woman explained that, while they used to be camel‐herders and still owned a large number of livestock, in 2004 they learned the value of cars and sold some of their animals to buy cars to support their trading activities in towns.

Petty trade by women to earn cash to cover their daily needs (such as oil, onions, salt and sugar) is very common. Women sell their agricultural produce, grasses or firewood they collect themselves or buy from others, plus woven items they have made. In Manzola, firewood is a vitally important source of cash for women, who have few other options. They collect it from nearby mountains and remote areas to meet their own needs and then younger girls sell what is left in neighbouring markets. In Doga, women sometimes face harassment from hostile livestock owners, so they travel to Chad to collect wood, where they risk being fined by the Forest Protection Administration. They consider this preferable compared with the risks of harassment in Sudan.

Among settled nomadic communities, uniquely male activities include herding livestock (cattle, camels and sheep), labour migration, trade, car driving, gold mining and military service. The latter was considered particularly lucrative, providing regular wages at five times the rate a teacher is paid, according to one women's FGD.

Regarding livestock, women are responsible for looking after the small stock (goats and some sheep) that remains closest to the villages, for milking and preparing and trading milk products. In El Kother, a nomadic community that was forced to relocate because of conflict, women described how trade activities had helped them increase their income and also create a trading relationship between them and other people in their new area, and so was doubly rewarding.

In the dry season, women engage in a variety of low‐paid day labour, most of it manual or menial. In most areas, brick‐making started recently—this was from 2010 in Manzola, following a decrease in wood and charcoal, as people were prevented from entering the forest to cut wood by individual livestock herders. Women are employed as day labour to make the bricks; men's work is to stack the bricks before firing.

**Figure 5:**
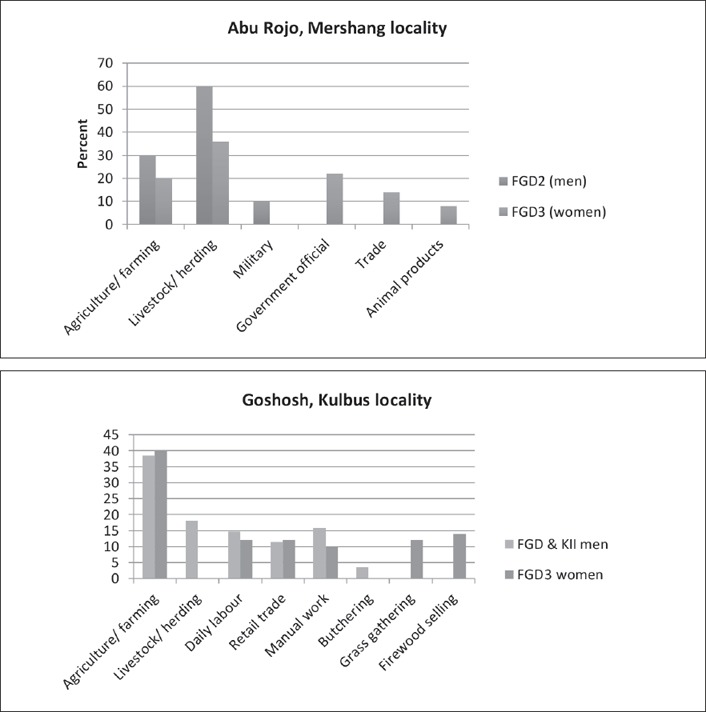
Livelihood activities identified by FGDs in South and West Darfur (Goshosh) **Source**: authors.

Importantly, women and men's FGDs gave slightly different accounts of their community's livelihood activities, with the biggest differences being that women tended to downplay livestock as a livelihood activity compared with men. Figure 5 shows an example of proportional piling exercises by men and women FGDs in the same village. In Goshosh, a mixed farming community, women ignored the contribution of livestock altogether. In Abu Rojo, they downplayed the contribution of livestock compared with men but emphasised animal products. Generally, women emphasised the plethora of marginal activities in addition to farming (grass gathering, selling firewood, petty trade, selling water, etc.). Manual work and day labour was important for both men and women.

The pattern of livelihood diversification and out‐migration of men has contributed to an increased workload for women in all communities, exacerbating the existing unequal division of labour. There has also been an impact on youth, with young men increasingly drawn to the towns for dry season work opportunities, to the gold mines and to the armed services.

### Shifts in the management of natural resources, and implications for farmer–herder relationships

Traditional systems for managing and accessing natural resources have fundamentally changed for both farming and pastoralist groups, and the integration that was common between livelihood systems earlier has been undermined.

A series of different conflicts have affected these communities and their livelihoods, leading to displacement, relocation to new areas, looting, raiding and loss of assets, combined with a deterioration in the relations between nomadic and farming communities, most visibly in the past 10–20 years linked with the conflict dynamics, but with deeper roots in changing land use practices following on from the famine of the early 1980s and subsequent transformation of livelihoods.

While conditions have improved, all communities expressed concerns. Former displaced communities complained about their loss of farmland and livestock, and, for some, continuing restrictions on accessing their farms or other natural resources. Pastoralist communities were concerned about the threat of livestock looting and armed robbery, which caused them to restrict their livestock migrations. While some communities, principally the settled nomadic communities, have emerged better off in terms of their livelihood assets after more than a decade of conflict, others have become impoverished, suggesting there are winners and losers. But this hides other disparities; among the nomads there is a dire lack of development, with far poorer educational and literacy levels and exceptionally high maternal mortality, partly linked to a lack of services.^3^ This lack of social development is a major source of grievance.

Almost all natural resources in Darfur, including water, cultivable land, forestry products, fodder and hay, now have an economic market value, and these market‐based systems co‐exist alongside customary institutions for managing land and other resources, generating an institutional plurality. Sales of sought‐after fertile land along *wadis* are common in communities serving urban markets. In Hashaba in South Darfur, small and medium urban investors have recently bought much of the cultivable land from small farmers, who now are working those same farms, not as owners but as day labourers.

In the past, crop residues and manure were shared freely. This system provided significant benefits for both herders and farmers and thus promoted good relations between them; livestock benefited from the residues at the beginning of the dry season as available pastures began to dry out. Farms benefited from the manure fertilising the soil, and pastoralist animals helped transport the produce from field to the grain store. This also provided opportunities for farmers and herders to exchange produce.

The *talaig* is the date set by the local tribal authorities when pastoralist livestock herds are permitted to enter farms after the harvest of rain‐fed sorghum and millet in order to graze on the stubble and crop residues. The setting of the *talaig* date is timed to coincide with completion of the harvest, when pastoralist herds are returning south from their northern rainy season pastures moving through the farming zone to grazing areas further south. The date of the *talaig* varies, occurring earlier (in December) during years of rain shortage and lean harvest and later (up to March) during years of better rains and plentiful harvest, which takes longer to gather in.

With time these practices have declined. These by‐products are now frequently collected by the owner for own use or sold as a source of income. Farmers reported that, since the outbreak of conflict in 2003, they had increasingly cleared crop residues for their own use or for sale, partly, they said, to avoid any problems with passing livestock herds trespassing on their farmland. Farmers use part of the residues for building and repairing their housing and fences or feeding their own animals. Alternatively, women sell crop residues on the market as a source of income to cover household expenses. Often, this involves crop residues being sold in nearby towns rather than recycled locally.

In Telehaya, West Darfur, a settled nomadic community, women explained that they collected manure in *'great quantities and put it in sacks on donkeys. The girls take it to the construction places or brick kilns to sell*'. According to one Telehaya women's FGD, selling manure contributes 90% of their cash income at the end of dry season (when it is easier to collect because of the concentration of livestock around water points).

This commoditisation of natural resources has contributed to increasing competition and conflict over former shared resources and increasing pressures on customary institutions intended to manage them.

The market price of crop residues fluctuates depending on the rainy season and the harvest. In good years residue prices are low, because there is increased supply and reduced demand for residues as a source of fodder as pasture is widely available and livestock herds are more likely to stay further north for longer. Conversely, when rainfall, harvests and pastures are poor, the supply of residues is much reduced, herds are forced to return south earlier and high demand pushes up prices. Thus, problems are more likely to occur between farmers and herders in drier years. Farmers frequently complained of livestock herders not respecting the *talaig* date; on the other hand, some farmers are removing all residues for their own use or sale, leaving nothing behind for the pastoralist livestock.

According to farmers, an added complication caused by the wider conflict is that herders are frequently heavily armed while the farmers are not. In the past, the local sheikh would mediate farmer–herder disputes, but, given recent conflict dynamics and the weapons carried by herders, they have limited power and authority over herders. Herders, on the other hand, argued that they had to carry arms to protect their herds from looting, which is a widely reported threat. The recently settled nomads in Telehaya who are farming also complained about damage to their crops caused by pastoralist herds, which indicates that farmer–herder conflicts do not necessarily correspond to ethnic conflicts between tribes with nomadic and farming identities.

For farmers, disputes with herders over livestock‐damaging crops were the major problem they faced at the time of the study. At least three of the five farming communities have taken steps to avoid problems with pastoralist herds by establishing Peaceful Co‐Existence or Farm Protection Committees, which include farmers, herders and sheikhs.

## Discussion

### Livelihood specialisation as an ‘adaptation'

In the Darfur region, rain‐fed farming and pastoralism have co‐evolved with conditions of extreme environmental variability, with each representing a successfully adapted livelihood specialisation. Important dimensions of integration between the two have played a key role in this success. Both individually and combined, these specialisations are examples of the way agricultural productivity in dry land environments can be improved by working with variability rather than against it (Krätli, 2015). Rainfall patterns vary between years and over decades, and during a single rainy season are unpredictable and uneven in space and time. There is a pronounced north–south rainfall gradient (with increasing annual rainfall from south to north) with a corresponding but reversed gradient in the concentration of nutrients, which means the more nutritious pastures are to be found in the sparser northern rangelands in the rainy season (Bremen and de Wit, 1983). Pastoralists and farmers take advantage of this variability in their seasonal migrations (Young et al., 2016) and in their farming strategies and real‐time adjustments in cropping and grazing strategies (Krätli, 2015). This accounts for their continuity as the dominant livelihood strategies in the region.

Community perceptions of seasonality and the corresponding livelihood strategies provide a flexible framework for understanding how communities manage their production in a context where the timing of seasons and activities within them varies. This seasonal framework also reveals the limits of this adaptation. While these systems are specialised to work with environmental variability, they cannot withstand persistent drought, which by definition is a lack of variability. As Ellis and Swift (1988) point out, a strategy for managing severe drought is to extend the scale of the system, for example by moving livestock outside the area hit by drought or by keeping grain reserves for longer than two to three years. Shocks that restrict the specialist strategies—for example conflict and insecurity restrict livestock mobility and farmers’ access to natural resources—are another example of the limits of this adaptation. Combinations of shocks are a further threat to this adaptation, as effects on livelihood systems are likely to be multiplied synergistically.

Seasonality and variability permeate almost every aspect of people's lives; even the relationships between herders and farmers vary seasonally, with risk of tensions and disputes peaking during the rain‐fed and dry season cultivation periods, when there is risk of crop damage by livestock, or blocking of livestock corridors by farms, for example.

### Differential effect of climate and conflict shocks

Pastoralist communities appear to have been less affected than farming communities by occasional droughts over the past 30 years. However, the severity of the drought impact is related to the persistence of the drought (over consecutive years), and also whether it is combined with other shocks. Similar observations have been made about pastoralist people and herds in Turkana, Kenya, where the authors concluded that a single year of drought was survivable (Ellis and Swift, 1988). Similarly, reflecting on Darfur, Morton (1993) points out, ‘Drought alone they could have dealt with’ (p. 4), but the challenges and shocks facing these specialist systems are far more complex and persistent than one‐off environmental shocks.

While Darfuri producers have developed strategies to manage extreme environmental variability, these are now combined with strategies to cope with other challenges. This long history of extreme variability and risk of drought and floods has combined with a series of economic, political and social change processes to exert pressures and impact on livelihood systems, producing transformational changes to the communities in this study and their livelihood systems.

Over time, communities have intensified and diversified their mix of livelihood activities in order to secure their subsistence and cash needs. While their specialisation is maintained as the basis of their production, management practices are changing (herd composition and the shift to sheep, continuous farming with no fallow periods, more restricted livestock mobility). In addition, both livelihood sub‐systems have diversified by introducing either farming or livestock herding, plus migration and trade. Among the poor, there is heavy reliance on a range of more marginal activities (day labour, collection and sale of manure, grass and firewood, charcoal‐ and brick‐making), which are predominantly done by women.

The unequal division of labour, which falls heavily on women, has been reported earlier, and it is argued provides the background for male out‐migration in search of wage labour elsewhere (Grawert, 1992). More than 20 years later, this gendered division of labour is amplified, which must limit the household's productivity and resilience in the longer term.

### Coping or maladaptation?

While these changes in livelihood strategies fit well with earlier frameworks that describe three processes of adaptation—agricultural intensification, livelihood diversification and migration (Hussein and Nelson, 1998; Scoones, 1998)—they also raise deeper issues of loss of integration between specialist systems. These are linked to increasing competition and illicit or maladaptive livelihood activities arising in connection with conflict and the war economy.

In the Darfur context, the ubiquitous use of the term ‘coping strategy', which originates from the earlier drought coping literature, ignores the potential harm these responses cause. Maladaptation may be a more appropriate term in this context; this implies these strategies are functionally linked to the conflict or war economy, or post‐conflict dynamics, which differentiates them from peacetime strategies. Where one person's coping strategy severely restricts the livelihood specialisation of another (such as by limiting their access to farmland or other natural resources or by restricting their livestock mobility or encroaching rangeland), this directly works against the fundamental strategy in the specialisation to make use of environmental variability. In his definition of sustainable livelihoods, Scoones (1998) took account of the impact of livelihood actions on the natural resource base and the livelihoods of others. Until more recently, there has been little attention to the way in which maladaptation undermines resilience (Barnett and O'Neil, 2010; IPCC, 2012; Ferguson and Brown, 2014; Levine, 2014), with little or no consideration in relation to conflict.

### Diversification, integration and increasing competition

The past 30–40 years has seen a process of increasing diversification at the household level, with pastoralists taking up farming while farmers are investing in livestock. Yet each lacks the specialist skills and experience of the other. Also, their motivations to farm or herd often differ, as these activities are additional to their predominant livelihood strategy. For example, farming supports pastoralism as it reduces the need to sell livestock to buy cereals and provides a source of crop residues to serve as fodder. In a drought year, a failed harvest is a valuable source of grazing for the pastoralist herd. Hence, while livelihood strategies may appear to have become more universal, the producer goals, skills and management practices vary enormously.

Farmers diversifying into livestock, especially sheep, reflects a wider trend driven by the increase in Sudan's export of sheep, which, as Abdul‐Jalil (2008) points out, has brought about competition with nomads. Similarly, the urbanisation of Darfur has led to an increase in purchased fruits and vegetables, leading to another trend of investment, in dry season irrigated horticultural activities in the *wadi* areas favoured for grazing by pastoralist herds in the dry season. These emerging new factors have increased competition between the specialist producers (Abdul‐Jalil, 2008).

Crop–livestock integration is commonly understood in the literature as mixed farming at the scale of the farm, and has been promoted as a way to increase the efficiency of production through agricultural intensification in sub‐Saharan Africa (Ellis and Galvin, 1994). This farm‐level focus conceals the ways in which integration has developed in dry land regions like Darfur, particularly as livestock mobility allows for multiple opportunities for integration, over time and across space (Krätli et al., 2015). This is well illustrated in this study by the former positive interactions between specialist producers, frequently described as a symbiotic relationship between farming and herding systems and a sharing of mutual benefits (Barth, 1973; Fadul, 2004; Young et al., 2013). At a range of scales, this allows for the integration of two specialist systems, with multiple benefits and little trade‐off in specialisation.

In addition to conflict, other factors have played a major role in undermining the relationships and former integration. These include: the diversification of livelihood activities, which results in all households seemingly practising the same few livelihood activities; market competition driving agricultural production and growth of dry season cash crops and investments in sheep; the commoditisation of shared natural resources, especially land, water, manure, fodder and crop residues; and the decline or failure of natural resource institutions that sustain these former symbiotic relationships.

The commercialisation of agriculture is reflected in Darfur's economy, which is underpinned by its agriculture sector, especially crops and livestock. In the past, agricultural production was increased by extending the cultivating area, and it is unclear if this option is still available without exacerbating the encroachment of rangeland or the blocking of livestock corridors. Increasingly, farmers are trying to intensify their production by including dry season cash crop production as well as rainy season subsistence production, and increasing their investments in irrigation and other inputs for the cash crops. Farmers in this study reported shrinking farm size and reduced soil fertility linked with continuous cultivation. The Ministry of Agriculture confirmed declining farm productivity, especially of millet and sorghum, over a 30‐year period, attributed to continuous cultivation, and the expansion of cereal production into increasingly marginal land, without use of fertilisers or crop rotation (Buchanan Smith et al., 2014).

Overall, livestock numbers are reported to be increasing (Behnke and Osman, 2010; Krätli et al., 2013), although among the former displaced communities in West Darfur livestock losses are considerable. Among livestock‐owning communities, the composition of herds is changing in response to the market demands for sheep and associated quick economic returns (Young et al., 2013, 2016).

There are multiple papers on how changing land use practices in Sudan, including the expansion and intensification of agriculture, together with the erosion of customary institutions, have brought pastoralists into conflict with farmers (Glover, 2005; Manger, 2005; Siddig et al., 2007; Abdul‐Jalil, 2008; Sulieman, 2015). This study provides a unique lens on the erosion of an important customary institution, the *talaig*, and the implications for farmer–herder relationships and livelihood resilience.

While the committees are focused on the problem of crop damage by livestock, clearly the tensions between farmers and herders have deeper roots and drivers. A long‐term trend is the expansion of rain‐fed farming (reducing the availability of rangeland) and the extension and expansion of dry season farming. Dry season farming involves cultivation of *wadi* soils, in precisely the area where pastoralist herds depend on shallow wells in the dry season, which again increases the risk of livestock damaging crops. Another long‐term trend is the decline in cooperative activities between farmers and herders and mutual benefits associated with the *talaig*, partly associated with the commoditisation of crop residues. Hence, there are long‐term processes that have undermined relationships between farmers and herders, which have been exacerbated and enflamed by conflict dynamics and especially the 2004 government counter‐insurgency tactics of mobilising militia and pitting them against the perceived rebel‐supporting communities. While for some communities this conflict is now in the past and they want to move on, there are inevitably others whose livelihoods and lives have radically changed for the worst.

The setting‐up of local committees to address the problems of weakened natural resource institutions reflects local recognition and commitment to addressing the problems arising from the rifts and loss of integration. A crucial step as part of this process lies in drawing in or engaging wider networks, including government, civil society and the international community, and developing a shared understanding (an approach advocated elsewhere (Cleaver, 2012)). This understanding needs to acknowledge the experiences of local people and their ability to manage variability through their livelihood specialisations, and also the inequitable resource relations that have evolved over the past decades as a result of conflict and wider social, economic and political processes.

## Conclusions

Darfuri producers, whether in farming or pastoralism, are specialised to take advantage of extreme environmental variability. The roots of resilience of Darfur livelihood systems rest in the continuity and integration of these livelihood specialisations, as part of a regional livelihood system. Despite this inherent resilience, Darfur communities remain vulnerable to the continuous stream of climate, conflict and other shocks that have affected them throughout their lifetimes. Their well‐rehearsed specialist strategies are now combined with newly needed strategies to cope with protracted conflict, insecurity and other shocks. Sometimes, the new strategies replace the specialist strategies or compensate for the impossibility of properly applying them. While these new strategies help people get by in the short term, they risk antagonising not only their own specialist strategies but also those of others.

While continuity in livelihood specialisations is still strongly evident, these specialisations have been undermined by the on‐going conflict and insecurity, with a loss of integration and even polarisation between them. In addition to the endless stream of shocks, demographic pressures combined with market forces have led to processes of commoditisation of natural resources, which can now all be bought and sold on the market. This has contributed to increasing competition and conflict over common property resources and increasing pressures on traditional institutions intended to manage them.

The future sustainability of farming and pastoralist production systems depends on their ecological sensitivity in managing environmental variability, and their integration and peaceful co‐management of natural resources, which is influenced by their institutional relationships across multiple levels. Humanitarian actors respond to humanitarian needs, which by definition target affected individuals, households and communities. In the Darfur context, this initially led to a stove‐piping approach that reinforced the polarisation between pastoralists and displaced farming communities. While this has been recognised to some extent, much remains to be done in relation to promoting policy and programme approaches that support the continuity and integration between livelihood specialisations.

## Acknowledgements

This study was funded by UK aid from the Department for International Development under its support to the programme Building Resilience in Chad and Sudan, led by Concern Worldwide. It was supported by staff from the State Ministry of Animal Resources and Agriculture, West Darfur, Al Massar organisation and research staff from the Universities of Zalingei, El Fasher, Nyala and Gadaref. Special thanks go to the reviewers of this paper and also to Saverio Krätli for his guidance and inputs in the initial stages of this study.

## Endnotes

1 Displacement tends to refer to the forced displacement of farming communities to internally displaced person camps, whereas the conflict‐related movement of nomadic communities is rarely described as forced displacement but involves involuntary relocation of entire communities as in the case of El Kother, North Darfur, in this study.

2 Desert sheep are the most popular but local breeds adapted to the wetter conditions further south are also increasing in popularity.

3 As revealed in the BRICS baseline survey in 2016.

## References

[disa12337-bib-0001] Abdul-Jalil, M.A. (2008) ‘Nomad-sedentary relations and the question of land rights in Darfur: from complementarity to conflict’. In Rottenberg R. (ed.) Nomadic-Sedentary Relations and Failing State Institutions in Darfur and Kordofan (Sudan). Orientwissenschaftlichen Zentrum der Martin-Luther-Universität Halle-Wittenberg, Halle.

[disa12337-bib-0002] Adger, W.N. (2000) ‘Social and ecological resilience: are they related?’. Progress in Human Geography. 24(3). pp. 347–364.

[disa12337-bib-0003] Barnett, J. and S. O'Neil (2010) ‘Maladaptation’. Global Environmental Change. 20 pp. 211–213.

[disa12337-bib-0004] Barth, F. (1973) ‘A general perspective on nomad-sedentary relations in the middle east’. In Nelson C. (ed.) The Desert and the Sown Nomads in the Wider Society. Institute of International Studies, University of California, Berkeley, CA pp. 11–21.

[disa12337-bib-0005] Behnke, R. and H.M. Osman (2010) The Contribution of Livestock to the Sudanese Economy. IGAD LPI Working Paper 01-12. IGAD Livestock Policy Initiative, Odessa Centre, Great Wolford.

[disa12337-bib-0006] Behnke, R.H. , I. Scoones , and C. Kerven (1993) Range Ecology at Disequilibrium: New Models of Natural Variability and Pastoral Adaptation in African Savannas. ODI and IIED, London.

[disa12337-bib-0007] Bousquet, F. , A. Botta , L. Alinovi , et al. (2016) ‘Resilience and development: mobilizing for transformation’. Ecology and Society. 21(3). pp. 1–18.27668001

[disa12337-bib-0008] Bradshaw, S. (2015) ‘Engendering development and disasters’. Disasters. 39(S1). pp. S54–S75. 25494957 10.1111/disa.12111

[disa12337-bib-0009] Bremen, H. and C. T. de Wit (1983) ‘Rangeland productivity and exploitation in the Sahel’. Science. 221(4618). pp. 1341–1347. 17758992 10.1126/science.221.4618.1341

[disa12337-bib-0010] BRICS (Building Resilience in Chad and Sudan) (2016) Baseline study findings. Concern Worldwide, World Agroforestry Center, Feinstein International Center, Al Massar.

[disa12337-bib-0011] Buchanan Smith, M. , A.J. Abdulla , A. Rahman , et al. (2014) Against the Grain: The Cereal Trade in Darfur. Feinstein International Center, Tufts University, Somerville, MA.

[disa12337-bib-0012] Cleaver, F. (2012) Development Through Bricolage: Rethinking Institutions for Natural Resource Management. Routledge, Abingdon.

[disa12337-bib-0013] De Waal, A. (2004) ‘Counter-insurgency on the cheap’. London Review of Books. 15 August.

[disa12337-bib-0014] Ellis, J. and K.A. Galvin (1994) ‘Climate patterns and land-use practices in the dry zones of Africa’. BioScience. 44(5). pp. 340–349.

[disa12337-bib-0015] Ellis, J.E. and D.M. Swift (1988) ‘Stability of African ecosystems: alternate paradigms and implications for development’. Journal of Range Management. 41(6). pp. 450–459.

[disa12337-bib-0016] Fadul, A.A. (2004) ‘Natural resources management for sustainable peace in Darfur’. In Environmental Degradation as a Cause of Conflict in Darfur. Conference Proceedings. University for Peace, Africa Programme, Addis Ababa and Khartoum.

[disa12337-bib-0017] Ferguson, B. and R. Brown (2014) ‘Transformation or maladaptation? Exploring the influence of desalination on Melbourne's water resilience’. https://resilience2014.sciencesconf.org/25080/document (last accessed on 13 January 2019).

[disa12337-bib-0018] Flint, J. and A. de Waal (2005) Darfur: A Short History of a Long War. Zed Books, New York.

[disa12337-bib-0019] Folke, C. (2006) ‘Resilience: the emergence of a perspective for social-ecological systems analyses’. Global Environmental Change. 16 pp. 253–267.

[disa12337-bib-0020] Glover, E.K. (2005) ‘Tropical dryland rehabilitation: case study on participatory forest management in Gedaref, Sudan’. Faculty of Agriculture and Forestry, University of Helsinki, Helsinki.

[disa12337-bib-0021] Grawert, E. (1992) ‘Impacts of male outmigration on women: a case study of Kutum/Northern Darfur/Sudan’. Ahfad Journal: Women and Change. 9(2). pp. 38–60. 12319274

[disa12337-bib-0022] Grawert, E. (2008) ‘Cross-border dynamics of violent conflict. The case of Sudan and Chad’. Journal of Asian and African Studies. 43(6). pp. 595–614.

[disa12337-bib-0023] Haaland, G. (1991) ‘Systems of agriculture in Western Sudan’. In Craig G. (ed.) The Agriculture of the Sudan. Oxford University Press, Oxford pp. 230–251.

[disa12337-bib-0024] Holling, C.S. (1973) ‘Resilience and stability of ecological systems’. Annual Review of Ecology and Systematics. 4 pp. 1–23.

[disa12337-bib-0025] Hussein, K. and J. Nelson (1998) Sustainable Livelihoods and Livelihood Diversification. IDS, Brighton.

[disa12337-bib-0026] IPCC (Intergovernmental Panel on Climate Change) (2012) Managing the Risks of Extreme Events and Disasters to Advance Climate Change Adaptation. Special Report of the IPCC. IPCC, Cambridge University Press, Cambridge.

[disa12337-bib-0027] Krampe, F. (2017) ‘Toward sustainable peace: a new research agenda for post-conflict natural resource management’. Global Environmental Politics. 17(4). pp. 1–8.

[disa12337-bib-0028] Krätli, S. (2015) Valuing Variability. New Perspectives on Climate Resilient Drylands Development. IIED, Rainfed Livestock Network, Revitalizing Rainfed Agriculture, Drylands Learning and Capacity Building Initiative for Improved Policy and Practice in the Horn of Africa, Peking University.

[disa12337-bib-0029] Krätli, S. , O.H. Eldirani , H. Young , et al. (2013) Standing Wealth. Pastoralist Livestock Production and Local Livelihood in Sudan. Feinstein International Center, Tufts University, UNEP, SOS Sahel Sudan, Ministry of Animal Resources Fisheries and Range, Nomad Development Council, Khartoum.

[disa12337-bib-0030] Krätli, S. , B. Kaufmann , H. Roba , et al. (2015) A House Full of Trap Doors: Identifying Barriers to Resilient Drylands in the Toolbox of Pastoral Development. Discussion Paper. IIED, London.

[disa12337-bib-0031] Le Masson, V. (2015) Gender and Resilience: From Theory to Practice. Working Paper. BRACED Knowledge Manager, ODI, London.

[disa12337-bib-0032] Levine, S. (2014) Assessing Resilience: Why Quantification Misses the Point. Humanitarian Policy Group, ODI, London.

[disa12337-bib-0033] Mamdani, M. (2009) Saviors and Survivors: Darfur, Politics, and the War on Terror. Pantheon Books, New York, NY.

[disa12337-bib-0034] Manger, L. (2005) ‘Understanding resource management in Western Sudan. A critical look at new institutional economics’. In Gausset Q. and Thomsen T.B. (eds.) Beyond Territory and Scarcity: Social, Cultural and Political Aspects of Conflicts on Natural Resource Management. The Nordic Institute of African Studies, Uppsala.

[disa12337-bib-0035] Matyas, D. and M. Pelling (2015) ‘Positioning resilience for 2015: the role of resistance, incremental adjustment and transformation in disaster risk management policy’. Disasters. 39(S1). pp. S1–S18. 25494954 10.1111/disa.12107

[disa12337-bib-0036] Mortimore, M. , with S. Anderson , L. Cotula , J. Davies , et al. (2009) Dryland Opportunities: A New Paradigm for People, Ecosystems and Development. IUCN, IIED and UNDP, Gland, Switzerland.

[disa12337-bib-0037] Morton, J.F. (1993) ‘Agricultural development in Darfur region, Sudan: with special reference to innovation, technical change and open access resources’. PhD, London University, London.

[disa12337-bib-0038] Osman, A.M.K.O. (2012) ‘Agricultural change, land and violence: an examination of the region of Darfur, Sudan’. PhD Dissertation, Tufts University, Boston, MA.

[disa12337-bib-0039] Osman, A.M.K. , H. Young , R.F. Houser , and J. Coats (2013) Agricultural Change, Land, and Violence in Protracted Political Crisis: An Examination of Darfur. Research Backgrounder. Oxfam America, Boston, MA.

[disa12337-bib-0040] Pyle, A.S. (1993) ‘Household vulnerability to famine: survival and recovery strategies among the Berti and Zaghawa migrants in northern Darfur, Sudan, 1982-1989’. Geojournal. 30(2). pp. 141–146.

[disa12337-bib-0041] Scoones, I. (1996) Living with Uncertainty. New Directions in Pastoral Development in Africa. ITDG, London.

[disa12337-bib-0042] Scoones, I. (1998) Sustainable Rural Livelihoods: A Framework for Analysis. Working Paper 72. IDS, Brighton.

[disa12337-bib-0043] Siddig, E.F.A. , K. El-Hanzi , B. Prato , et al. (2007) Managing Conflict Over Natural Resources in Great Kordofan, Sudan. International Food Policy Research Institute, Washington D.C.

[disa12337-bib-0044] Sulieman, H. and H. Young (2018) Understanding Change: Transforming Pastoral Mobility in West Darfur. Building Resilience in Chad and Sudan, Concern Worldwide, Feinstein International Center, Tufts University, Medford, MA.

[disa12337-bib-0045] Sulieman, H.M. (2015) ‘Grabbing of communal rangelands in Sudan: the case of large scale mechanized rain-fed agriculture’. Land Use Policy. 47 pp. 439–447.

[disa12337-bib-0046] UN (United Nations) (2013) 2013-2019 Developing Darfur: A Recovery and Reconstruction Strategy. Pursuant to Article 31 of the Doha Document for Peace in Darfur.

[disa12337-bib-0047] Walker, J. and M. Cooper (2011) ‘Genealogies of resilience: from systems ecology to the political economy of crisis adaptation’. Security Dialogue. 42(2). pp. 143–160.

[disa12337-bib-0048] Young, H. , A. Osman , Y. Aklilu , et al. (2005) Darfur – Livelihoods under Siege. Feinstein International Famine Center, Tufts University, Medford, MA.

[disa12337-bib-0049] Young, H. , H. Sulieman , R. Behnke , et al. (2013) Pastoralism in Practice: Monitoring Livestock Mobility in Contemporary Sudan. Feinstein International Center, Tufts University, Somerville, MA.

[disa12337-bib-0050] Young, H. , R. Behnke , and S. Robinson (2016) Risk, Resilience and Pastoralist Mobility. Feinstein International Center, Tufts University, Medford, MA.

